# Plunging Ranula: A Case Report of a Rare Late Complication After Tongue Cancer Surgery

**DOI:** 10.7759/cureus.22423

**Published:** 2022-02-21

**Authors:** Anshuman Kumar, Suhani Ghai, Garima Rawat

**Affiliations:** 1 Surgical Oncology, Dharamshila Narayana Superspeciality Hospital, New Delhi, IND; 2 Pathology, Dharamshila Narayana Superspeciality Hospital, New Delhi, IND

**Keywords:** case report, head and neck surgery, neck dissection, glossectomy, plunging ranula, ranula

## Abstract

A plunging ranula is a benign cystic lesion in the neck formed due to mucin extravasated from a salivary gland, most commonly the sublingual gland. Ranulas have been described in association with congenital anomalies, trauma, diseases of the sublingual gland, and HIV; however, rarely, they may result as a complication of various oral and neck surgeries. Here, we report a rare case of plunging ranula that developed in an elderly male as a sequalae to surgery for tongue cancer. The patient had undergone a partial glossectomy with supra-omohyoid neck dissection for tongue carcinoma and nine months later presented with cystic swelling on the floor of the mouth that was followed by neck swelling. It was treated successfully by excision, and the histopathology confirmed the diagnosis of ranula. We postulate that the tongue cancer surgery could have caused an inadvertent injury to the ducts of the sublingual salivary gland and mylohyoid muscle, leading to the development of a plunging ranula. Our case reiterates that surgeons need to be aware of the anatomy of the submandibular and submental region to avoid any surgical trauma to the sublingual and submandibular glands and their ducts along with the associated mylohyoid muscle.

## Introduction

Ranula is a benign cystic lesion formed due to mucin extravasated from a salivary gland, most commonly the sublingual gland. It is of two types: oral ranula and plunging ranula. The oral ranula is located on the floor of the mouth and is secondary to mucus extravasation that pools in a form of a cyst superior to the mylohyoid muscle. The plunging or cervical ranula is rarer than the oral ranula and presents as a neck swelling as a result of mucus extravasation that occurs along the fascial planes of the neck [1]. Ranulas usually occur in the pediatric age group and young adults, with the peak frequency in the second to third decade [2]. The etiology of ranula is usually unknown, but it has been described in association with congenital anomalies, trauma, diseases of the sublingual gland, and HIV [3]. Here, we report a rare case of plunging ranula that occurred in an elderly male as a sequalae to surgery for tongue cancer.

## Case presentation

A 69-year-old male presented to the outpatient department (OPD) of Surgical Oncology, with the chief complaint of painless swelling in the midline of the neck for three months. The patient, with a comorbid history of hypertension, was a follow-up case of carcinoma of the right lateral side of the tongue, which was operated on three years ago. At that time, the patient had presented with a nonhealing ulcer on the ventral surface of the tongue laterally following dental extractions due to sharp teeth. The incision biopsy from the lesion had shown verrucous keratosis with moderate keratinizing dysplasia. The patient underwent a right partial glossectomy with right extended supra-omohyoid neck dissection with anatomical closure. The histopathological examination of the intraoperative specimen showed a well-differentiated squamous cell carcinoma (superficially invasive) with negative margins. The lymph nodes were not involved. There was no perineural or lymphovascular invasion, and no other structures were infiltrated. After reviewing the histopathological report (pT1,N0,Mx), the patient was not administered any radiotherapy or chemotherapy.

Nine months after the surgery, the patient presented with a painless swelling on the floor of the mouth, which was approximately 1 cm in size, mobile, and soft. The swelling was compressible and used to subside spontaneously after eating food or applying pressure. Intraoral examination revealed a single, non-tender, cystic, pale white, fluctuant swelling on the floor of the mouth on the right side underneath the tongue. It was diagnosed as a case of postoperative oral (sublingual) ranula, and the patient was kept on regular follow-up as the patient did not want any active surgical intervention at that time.

**Figure 1:**
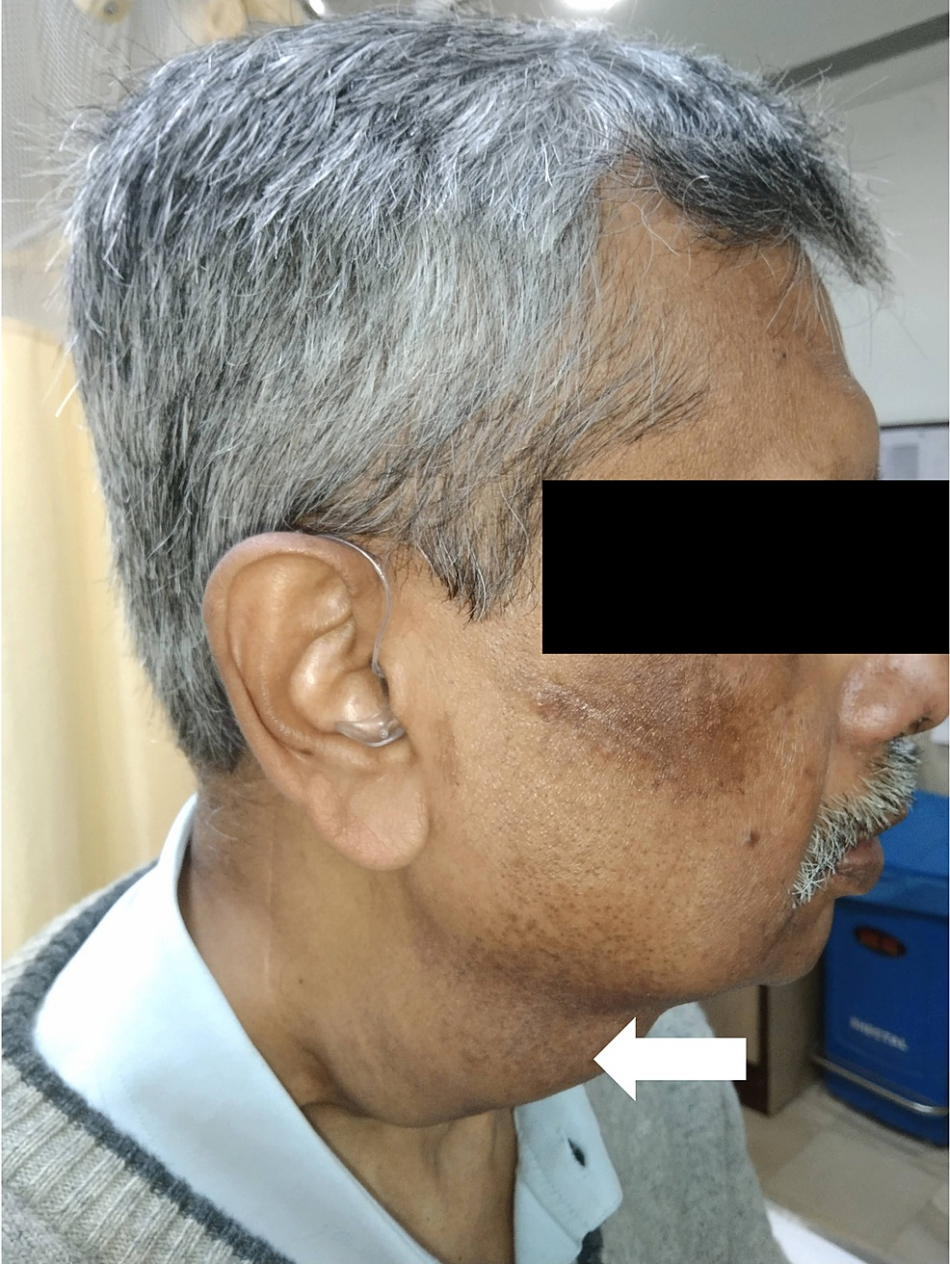
Visible swelling in the neck. Necessary informed consent was obtained from the patient.

About three to four months before presentation, the patient noticed that his swelling of the floor of the mouth disappeared spontaneously, but another swelling in the middle of the neck appeared, which was round, fluctuant, and non-tender, and did not disappear or reduce on eating food or applying pressure. The swelling was initially mobile, but with time, it progressively enlarged and became fixed. At the time of presentation to our OPD, it was about 5 cm in diameter (Figure 1).

The physical examination revealed the presence of a painless, soft, cystic, bulging mass extending from the right submandibular region to the cervical region.

**Figure 2:**
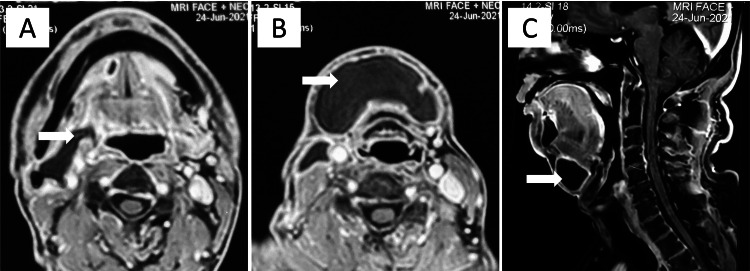
MRI with contrast of the face and neck showing (A) a non-enhancing thin-walled cystic lesion occupying the right submandibular region and continuing anteriorly into the sublingual space as the so-called “tail sign” (arrow); (B) the cystic lesion extending inferiorly into the submental space (arrow); and (C) submental cystic lesion (arrow) on sagittal section.

The MRI of the face and neck with contrast revealed a non-enhancing, thin-walled cystic lesion with clear fluid contents in the right submandibular and submental regions insinuating within the sublingual and parapharyngeal spaces suggestive of plunging ranula. There was no evidence of any recurrence of cancer (Figure 2).

**Figure 3:**
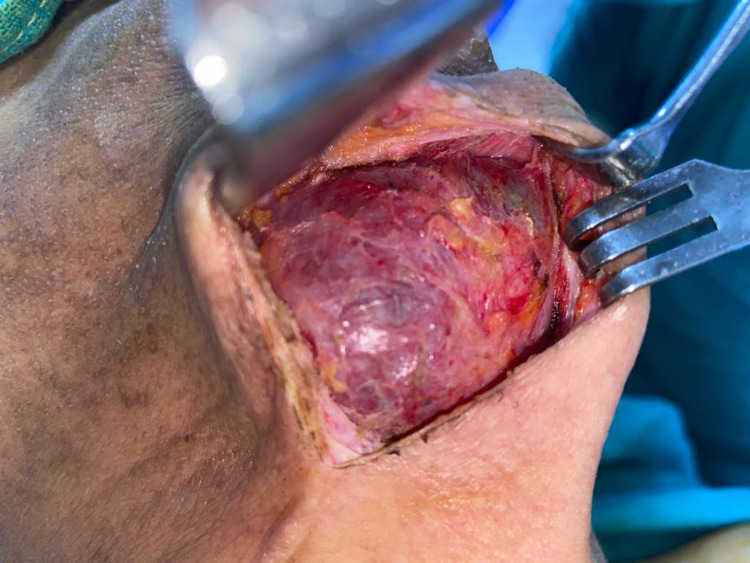
Intraoperative picture of the ranula being excised.

The patient underwent successful surgical excision of the ranula along with the sublingual gland under general anesthesia. The ranula was approached through a cervical incision placed in the neck. The preoperative finding was the presence of a multi-loculated cyst in the submental and submandibular region extending laterally on the right side into the parapharyngeal space (Figure 3).

**Figure 4:**
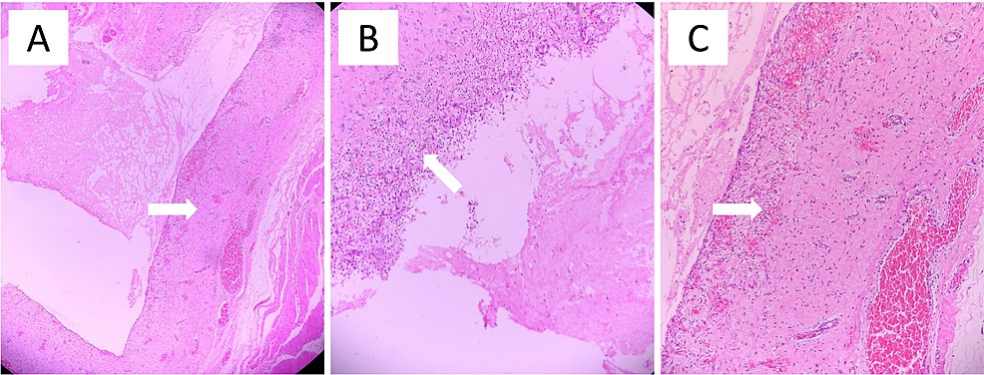
Pictomicrograph showing (A) ruptured mucin-filled cystic space (arrow) (H&E, 40×); (B) fibromuscular cyst wall (arrow), hemorrhage, and inflammatory cells (H&E, 100×); and (C) muciphages surrounding the mucin-filled area (H&E, 400×).

By careful layerwise dissection, the thin wall of the ranula was separated from the surrounding tissues. The ranula was excised in toto and sent for histopathological examination. The sublingual gland was approached and removed through an intraoral incision placed over the floor of the mouth. The ranula cyst contained mucoid material. Histopathology showed mucin-filled cystic space with fibromuscular cyst wall confirming the diagnosis of plunging ranula (Figure 4).

The surgical drain was removed in three days, and the patient was discharged by the fifth day. There was no postoperative infection. The patient was put on follow-up and showed complete healing of the surgical site with no recurrence of ranula at six months of follow-up.

## Discussion

The development of plunging ranula as a sequalae to tongue cancer surgery is an extremely rare occurrence, and to the best of our knowledge, this is the third such case described in the world literature. Dietrich et al. from Greece in 2011 had described a case of postoperative plunging ranula in a 45-year-old male with squamous cell carcinoma of the tongue that was treated with excision of the oral lesion with bilateral supra-omohyoid neck dissection and defect closure by left myocutaneous platysma flap [4]. Ten months postoperatively, the patient presented with a sublingual plunging ranula. The authors speculated that the ranula resulted from injury to the ducts of the sublingual gland during supra-omohyoid neck dissection [4]. The second case was reported by Xiong et al. from China who described a 45-year-old female who developed oral ranula eight months after hemiglossectomy, radical neck dissection, and reconstruction with vascularized radial forearm flap for tongue cancer [5]. Our case had a similar history of the development of ranula nine months after right partial glossectomy with right extended supra-omohyoid neck dissection for carcinoma of the right lateral side of the tongue. However, our case had certain notable differences from the previously described two cases. Our patient was an elderly, 69-year-old male who initially developed an oral (sublingual) ranula nine months after cancer surgery. Then, after a few months, his oral ranula swelling spontaneously disappeared, and he developed a plunging ranula without the oral component. This spontaneous conversion of an oral ranula into a cervical ranula has been rarely reported previously [2].

Ranulas may develop either as a result of a ruptured main duct of a salivary gland or from ruptured acini following ductal obstruction. This obstruction is often due to a mucus plug or sialolith; however, ductal stenosis, ductal hypoplasia or agenesis, chronic infection, or inflammation with periductal scarring, trauma, and neoplasia are other causes of ranula formation. In our case, we speculate that the partial glossectomy surgery could have caused an inadvertent injury to the sublingual salivary gland or its ducts, leading to the development of the oral ranula. The sublingual glands consist of a collection of 15-30 small, elongated glands located beneath the tongue, bordered medially by the genioglossus muscle of the tongue and laterally by the mandible. Secretions drain into the oral cavity by minor sublingual ducts, of which there are 8-20 ducts per gland, opening out onto the sublingual folds. Injury to the ducts of the sublingual gland can cause scar tissue formation and contracture that can seal and obstruct the gland, resulting in ranula [6,7].

A plunging or diving ranula occurs when an oral ranula bursts, with extravasation of its contents into the submandibular and submental space, either through the posterior free border of the mylohyoid or through a defect in the mylohyoid muscle (known as mylohyoid boutonniere). The herniated portion of the cyst leaves behind a taillike portion within the sublingual space, forming the pathognomonic “tail sign” [8]. Our case also had a similar sequence of events when his oral ranula vanished and he developed a plunging ranula. The mylohyoid muscle is considered the diaphragm of the floor of the mouth; however, it is not a strict anatomical barrier between the floor of the mouth and the neck. A hiatus or dehiscence in the mylohyoid muscle has been noted in 36%-45% of people [9]. Iatrogenic injury to the mylohyoid muscle during supra-omohyoid neck dissection may also create mylohyoid dehiscence, leading to plunging ranula. During supra-omohyoid neck dissection, the lymph nodes are separated from the anterior belly of the digastric and mylohyoid. A key step is the removal of lymph nodes located lateral to the mylohyoid muscle by retracting the anterior belly of the digastric muscle. The mylohyoid muscle is retracted anteriorly to expose the nerves and ducts in the region. The submandibular duct and ganglion are also divided [10]. Thus, many surgical steps involve the manipulation of the submandibular gland, mylohyoid muscle, and other associated structures in the region. Injury to these structures due to iatrogenic causes may predispose the patient to develop a sublingual or plunging ranula.

Many other surgical procedures have also been implicated in the formation of plunging ranula. It has been reported as a rare complication after bilateral submandibular duct transposition [11] and intraoral removal of submandibular gland sialolith [12]. Ranulas have also been reported as complications after intraoral surgery for submandibular duct relocation, ligation for treatment of sialorrhea, and dental implant surgery [13-17]. Thus, ranula formation after surgical procedures has been reported sporadically, but plunging ranula after tongue cancer surgery is an extremely rare finding.

## Conclusions

Although rare, plunging ranula formation may result as a complication of various oral and neck surgeries. Our patient, who developed ranula nine months after tongue cancer surgery, highlights that surgeons need to be careful during glossectomy and neck dissection to avoid any surgical trauma to the sublingual and submandibular glands and their ducts along with the associated mylohyoid muscle. In addition, patients must be kept on regular follow-ups, as ranula formation may occur many months after tongue cancer surgery.
